# Cadaverine’s Functional Role in Plant Development and Environmental Response

**DOI:** 10.3389/fpls.2016.00870

**Published:** 2016-06-21

**Authors:** Amy L. Jancewicz, Nicole M. Gibbs, Patrick H. Masson

**Affiliations:** ^1^Program in Cellular and Molecular Biology, Laboratory of Genetics, University of Wisconsin–Madison, Madison, WIUSA; ^2^Program in Plant Breeding and Plant Genetics, Laboratory of Genetics, University of Wisconsin–Madison, Madison, WIUSA

**Keywords:** polyamine, cadaverine, metabolism, plant development, root architecture, stress response, rhizosphere, phyllosphere

## Abstract

Cadaverine derives from lysine in a pathway that is distinct from that of the other well-characterized ornithine- or arginine-derived polyamines. Despite a multitude of studies in bacterial systems, cadaverine has garnered little attention in plant research. Nonetheless, many plants have been found to synthesize it. For instance, the *Leguminosae* have been shown to produce cadaverine and use it as a precursor in the biosynthesis of quinolizidine alkaloids, secondary metabolites that are involved in insect defense and also display therapeutic pharmacological properties. Cadaverine is also present in the environment; it can be produced by rhizosphere and phyllosphere microbes. Markedly, exogenous cadaverine application causes alterations in root-system architecture. Previous research suggests cadaverine has a role in stress response, with groups reporting an increase in content upon exposure to heat, drought, salt, and oxidative stress. However, data regarding the role of cadaverine in stress response remains conflicted, as some plant systems show enhanced tolerance to stresses in its presence, while others show increased sensitivity to the same stresses. In this review, we summarize recent findings on the role of cadaverine in plant growth, development, and stress response. We also address the possible roles rhizosphere and phyllosphere microbes may play in the delivery of exogenous cadaverine near plant organs, and discuss our current understanding of the molecular pathways that contribute to cadaverine homeostasis and response in plants.

## Introduction

Initially identified as a lysine decomposition product in organic matter, cadaverine, or 1,5-pentanediamine, is found ubiquitously in the environment. Cadaverine, from the word, cadaver, is often associated with decaying matter and is one of the components that gives carrion its distinctive smell. Cadaverine functions in a multitude of cellular processes critical to living organisms. In *Escherichia coli*, cadaverine is used to mediate acid stress (Haneburger et al., 2012), and the deathly odor of cadaverine provides behavioral cues to animals (Rolen et al., 2003; Hussain et al., 2013). In plants, it has been reported to contribute to plant growth and development, cell signaling, stress response, and insect defense. The regulation of these diverse processes is critical for plant fitness in natural ecosystems, and also for healthy crop production.

This minireview highlights contributions to the understanding of cadaverine’s functional role in plant development and environmental response by focusing on cadaverine’s biosynthesis and metabolism, its impact on plant growth and development, its potential contribution to plant-microbe interactions, and its role in stress response.

## Cadaverine Biosynthesis and Conjugation

**FIGURE 1:**
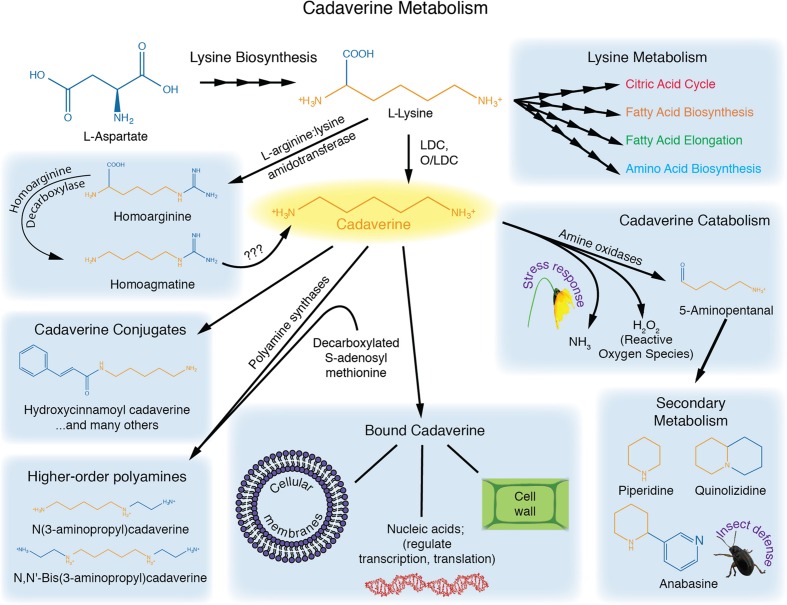
**Cadaverine metabolism.** Lysine serves as a precursor for cadaverine, and is critical for fatty acid metabolism, the citric acid cycle, and amino acid synthesis. Cadaverine can be conjugated to phenolics, or used to construct higher-order polyamines. Cadaverine can also be oxidized, or converted to quinolizidine alkaloids. Bound cadaverine may affect cell-wall properties, membrane stability, gene expression and nucleic acid stability.

Cadaverine is at the nexus of several biochemical pathways (**Figure 1**). Its main precursor is lysine, which is decarboxylated primarily by a lysine decarboxylase (LDC) found in the chloroplast (Schoofs et al., 1983; Wink, 1992; Bunsupa et al., 2012a). In some plants, such as *Lathyrus sativus*, cadaverine also appears to be synthesized from homoarginine via homoagmatine (reviewed in Bagni and Tassoni, 2001).

Endogenous cadaverine has been detected in several plant species, including wheat, rice, corn, and legumes (Felix and Harr, 1987), (reviewed in Tomar et al., 2013a). However, its concentration varies widely between species, organs, and even between different developmental stages of the same plant (Felix and Harr, 1987). In fact, some plants, such as *Arabidopsis thaliana*, may be deprived of a functional LDC, and have been reported to lack cadaverine. Moreover, enzymes previously annotated as putative LDCs in rice and *Arabidopsis* have instead been found to function in cytokinin biosynthesis (Kurakawa et al., 2007; Tokunaga et al., 2011).

A few dual-function enzymes, known as ornithine/lysine decarboxylases (O/LDCs), can accept both lysine and ornithine as substrates, thereby producing putrescine and cadaverine, respectively (Bunsupa et al., 2012a). In *Leguminosae*, the amount of cadaverine or putrescine synthesized by these enzymes appears to be regulated by substrate availability (Bunsupa et al., 2012a). O/LDCs are localized to the chloroplast, whereas the single-function ODC enzyme is localized to the cytosol, potentially allowing for compartmentalization of putrescine and cadaverine in plant cells (Voigt et al., 2000).

Most studies investigating cadaverine content in *Arabidopsis thaliana* have reported levels below detection (Bunsupa et al., 2012a; Liu et al., 2014; Strohm et al., 2015). Only one study has reported endogenous cadaverine ranging from 5 to 30 nmol g^-1^ FW in this species (Shevyakova et al., 2001). The discrepancy between these studies may reflect differences in growth conditions, with differential effects on cadaverine metabolism and/or conjugation. Indeed, cadaverine concentration is frequently reported only for the free form. However, a large fraction of cadaverine appears to be either in a bound form (to cell wall, membranes, or nucleic acids), or conjugated to small molecules such as phenolics, and to proteins (reviewed in Bagni and Tassoni, 2001). For instance, in tobacco, 90% of the polyamine pool was found in the conjugated form (Torrigiani et al., 1987), and a hydroxycinnamoyl transferase enzyme was identified and purified, which conjugates cadaverine, along with putrescine and diaminopropane, to caffeoyl-, cinnamoyl-, feruloyl-, sinapoyl-, and p-coumaroyl-CoA acceptors (Negrel et al., 1992). Such conjugates are important because they contribute to wall-polymer crosslinking. Similarly, in lupin, free cadaverine was undetectable while conjugated cadaverine was reported to be 4–13 nMol g^-1^ FW (Bunsupa et al., 2012a). Considering these observations, it will be important to test cadaverine levels in all polyamine pools to evaluate the possible existence of a cadaverine anabolic pathway in *Arabidopsis*.

Cadaverine is also a precursor for higher-order polyamines in plants. *S*-adenosyl methionine (SAM) is known to donate an *N*-aminopropyl group to cadaverine to form 3-aminopropylcadaverine and NN′-bis(3aminopropyl)cadaverine (Igarashi et al., 1986). Little is known about the function of these derived molecules.

## Cadaverine Catabolism and Conversion to Alkaloids

The catabolism of cadaverine is facilitated by amine oxidases, which use oxygen, water, and cadaverine to form 5-aminopentanal, ammonia, and hydrogen peroxide (Cona et al., 2006; Bunsupa et al., 2012b). Hydrogen peroxide is a free radical that, among other functions, can serve as a stress signaling molecule, while ammonia acts as a nitrogen source and may modulate salt tolerance in some species (Cona et al., 2006; Bunsupa et al., 2012b; Meng et al., 2016).

In *Leguminosae* and several other plants, 5-aminopental serves as a critical substrate for the synthesis of secondary metabolites called quinolizidine alkaloids, which are involved in insect defense responses (Wink, 1992; Yokota Hirai et al., 2000; Cona et al., 2006; Bunsupa et al., 2012a,b). These alkaloids are produced in the chloroplast and protect the plants against herbivorous insects, either by acting as a deterrent to feeding, or resulting in toxicity for the insect by affecting protein biosynthesis, membrane stability, or DNA and RNA processes (Wink, 1992, 2010; Bunsupa et al., 2012a).

Interestingly, some quinolizidine alkaloids, like lobeline, have been used as pharmaceuticals to treat central nervous system disorders and addiction by regulating dopamine uptake and release, and acetylcholine receptor modulation (Dwoskin and Crooks, 2002; Bunsupa et al., 2012b; Kaniakova et al., 2014). Alzheimer’s disease has also been treated using huperzine A, a quinolizodine-derived alkaloid that serves to increase acetylcholine levels by acting as a selective inhibitor of acetylcholinesterase (Bai et al., 2000; Bunsupa et al., 2012b).

## Is Environmental Cadaverine Taken Up by Plants?

Regardless of whether a given plant engages in *de novo* cadaverine biosynthesis, it is possible for this plant to take up cadaverine from its environment and use it for downstream metabolism and signaling. Indeed, maize seedlings were reported to take up exogenous cadaverine, putrescine, and paraquat (PQ, a broad-spectrum herbicide with structural similarity to polyamines) with similar kinetic profiles, and competition assays suggested a common plasmalemma transporter for these molecules (Hart et al., 1992). Similarly, soybean and rice were shown to take up cadaverine (Cassán et al., 2009; Ohe et al., 2010).

Until recently, the well-characterized polyamine transporters in plants, including members of the L-Amino acid Transporter family (LAT proteins, also named Polyamine Uptake Transporters (PUTs)) and Organic Cation Transporters (OCTs), had been primarily investigated for function in the transport of putrescine-derived polyamines or other organic cations (Mulangi et al., 2011; Fujita et al., 2012). OCT1, on the other hand, was suggested to function as a cadaverine eﬄux transporter because *oct1* knockout plants are known to display increased sensitivity to cadaverine (Strohm et al., 2015), and because spermine synthase over-expressing plants respond to cadaverine by increasing *OCT1* expression, possibly to eliminate excessive polyamine from the cells (Sagor et al., 2016).

These results are important because they suggest a contribution of two plasma membrane-associated proteins in cadaverine transport in plants. However, it will be critical to confirm these observations by verifying the cadaverine-transport activity of both proteins in heterologous expression systems. It will also be important to investigate other members of the AtLAT and OCT families for potential function in cadaverine transport.

## Do Microbes Generate Environmental Cadaverine for the Plant?

In the laboratory, plants are often grown in sterile conditions, or with limited access to their normal complement of microbes. Yet, in nature, protozoa, fungi, algae, and bacteria associate very intimately with shoot or root tissues, forming the phyllosphere and rhizosphere, respectively. Many microbes also colonize the internal spaces within plant organs, forming the endosphere (reviewed in de Vrieze, 2015, and in Schlaeppi and Bulgarelli, 2015), (Kuiper et al., 2001; Cassán et al., 2009; Lavizzari et al., 2010; Lundberg et al., 2012; Di Martino et al., 2013; Lebeis et al., 2015). These intimate associations between microbes and plants allow bidirectional signal exchange between organisms. They form incredibly complex biological networks that contribute to plant fitness, productivity and environmental responses (Ozawa et al., 2009; Pangesti et al., 2013).

Several microbes in the phyllosphere have been shown to produce cadaverine, sometimes at high concentrations. One study in spinach leaves detected 240 different bacterial isolates capable of producing cadaverine (Lavizzari et al., 2010). The rhizosphere also contains microbes that provide cadaverine for the plant, with downstream effects on stress protection and biomass production. For instance, rice seedlings inoculated with the cadaverine-producing bacterium *Azospirillum brasilense*, accumulated cadaverine in their tissues (in the pM range) (Cassán et al., 2009). Interestingly, rice treated with either cadaverine alone, or with *Azospirillum brasilense* culture, displayed gains in fresh and dry weight, and decreased sensitivity to osmotic stress, relative to controls (Cassán et al., 2009). These findings suggest a role for rhizosphere symbiont-synthesized cadaverine in the regulation of plant growth and stress mitigation. However, to solidify these findings, it will be important to determine the level of cadaverine in untreated rice seedlings. It will also be important to verify the inability of cadaverine-defective *Azospirillum* mutants to modulate plant sensitivity to stress.

## Cadaverine Modulates Plant Development

Alterations in cadaverine concentration within a plant, either caused by environmental stimulation of synthesis or through exposure to exogenous cadaverine, have been shown to induce morphological changes in a wide array of species, including *Arabidopsis*, rice, soybean, and Scots pine (Gamarnik and Frydman, 1991; Niemi et al., 2002; Cassán et al., 2009; Campestre et al., 2011; Liu et al., 2014; Strohm et al., 2015). **Table 1** summarizes reports of developmental responses to exogenous cadaverine.

**Table 1:** Exogenous application of cadaverine induces morphological changes.

Species	Treatment conditions	Result of cad treatment	[Cad]	Percent change
*Arabidopsis thaliana^1^*	Germinated and grown on Cad for 2 weeks	Primary root growth inhibition	2 mM	Not quantified
*Arabidopsis thaliana^2^*	Germinated and grown on Cad for 6 days	Primary root growth inhibition	100 μμM	2–65%*^A^
			500 μM	25–70%*^A^
		Change in lateral root branching	100 μM	-20–630%*^A^
			500 μM	5–850%*^A^
		Change in horizontal growth index	100 μM	10–70%*^A^
			500 μM	30–70%*^A^
		Change in straightness	100 μM	-2–10%*^A^
			500 μM	-10–20%*^A^
*Brassica juncea^3^*	Sown on filter paper with Cad solution	Increased germination after 24 h	1 mM	5%*
	Watered with Cad solution for 7 days	Decreased fresh weight	1 mM	15%*
*Glycine max^4^*	Germinated and grown on Cad for 5 days	Enhanced lateral root development	1 mM	Not quantified
*Glycine max^5^*	Germinated on soil and watered with Cad for 7 days	Hypocotyl elongation	25 μM	30% *
			50 μM	40%*
			100 μM	20%*
*Hordeum vulgare^6^*	Germinated and grown on Cad for 7 days	Increased coleoptile length	10 μM	21%
		Increased fresh weight	10 μM	4%
		Increased radicle number	10 μM	25%
	Cad pretreatment for 7 days then moved to soil until day 20	Increase in adaxial epidermis cells number	10 μM	34%
		Increase in abaxial epidermis cells number	10 μM	27%
		Increased distance between vascular bundles	10 μM	10%
		Increase in stomatal length	10 μM	27%
		Increased stomatal width of the abaxial leaf	10 μM	3%
*Oryza sativa^7^*	Cad applied on leaf surface of 2 day-old seedlings and grown for 7 days after treatment	Increased root fresh weight	1 nM	33%*^B^
			1 μM	44%*^B^
		Increased shoot fresh weight	1 nM	24%*^B^
			1 μM	22%*^B^
		Increased root dry weight	1 nM	29%*^B^
			1 μM	30%*^B^
		Increased shoot dry weight	1 nM	14%*^B^
			1 μM	22%*^B^
*Pinus sylvestris^8^*	Hypocotyl cuttings from 17 day old seedlings cultured 4 weeks in Cad	Decrease in root formation	500 μM	10%*^C^
*Vicia faba^9^*	Guard cell protoplasts from 3 week old plants treated with Cad	Inhibition of potassium influx	1 mM	37%*
		Decrease in stomatal pore size	1 mM	71%*

Summary of morphological changes induced by exogenous cadaverine treatment. Effect of cadaverine treatment was standardized to percentage for all studies. *Indicates approximate value when raw data was unavailable. (A) Results are presented as a range of values; the effect of cadaverine is dependent on natural accession studied. (B) No statistical test of significance. (C) Incomplete statistics. References: (1) Liu et al., 2014; (2) Strohm et al., 2015; (3) Tomar et al., 2013b; (4) Gamarnik and Frydman, 1991; (5) Campestre et al., 2011; (6) Çavuşoǧlu et al., 2007; (7) Cassán et al., 2009; (8) Niemi et al., 2002; (9) Liu et al., 2000.

Early functional studies were carried out in soybean seedlings, a species that produces relatively high amounts of cadaverine. In this system, exogenous cadaverine caused a decrease in primary root growth and an increase in lateral root branching (Gamarnik and Frydman, 1991). Similar observations were made in *Arabidopsis thaliana*, where alterations in primary root growth stemmed from a decrease in both cell division and cell elongation (Strohm et al., 2015).

Recently, it was suggested that cadaverine regulates root development and stress response by inducing spermine accumulation (Liu et al., 2014). In this study, *spms* and *pao4-1* mutants were found to display resistant and hypersensitive root-growth responses to cadaverine, respectively, compared to wild type. Cadaverine treatment promoted putrescine and spermine accumulation while reducing spermidine content. On the other hand, spermine was also shown to modulate plant’s sensitivity to cadaverine, as discussed earlier in this review (Sagor et al., 2016). Taken together, these initial studies are exciting because they begin to explore possible crosstalk between the cadaverine and putrescine-derived pathways in the modulation of root growth.

Reactive oxygen species (ROS) may also contribute to root-growth responses to cadaverine. Indeed, ROS were previously shown to play important roles in root development and stress response (Neill et al., 2002). As discussed earlier, cadaverine breakdown by amine oxidases primarily associated with the root apoplast leads to the production of hydrogen peroxide (Gamarnik and Frydman, 1991; Torrigiani and Scoccianti, 1995; Planas-Portell et al., 2013), a signaling molecule known to function at multiple levels in plant cells, including regulation of gene expression, DNA repair, cell-wall cross-linking, and programmed cell death (reviewed in Neill et al., 2002). Importantly, treating plants with hydrogen peroxide leads to primary root growth inhibition (Dunand et al., 2007). Analysis of ROS generation after spermidine treatment in *Arabidopsis* revealed a specific ratio of hydrogen peroxide to superoxide dictated the root growth phenotype characteristic of spermidine response (Andronis et al., 2014). By analogy, cadaverine may be partially acting through ROS to induce phenotypic changes, although more work is needed to test this contention.

Loci that contribute to root-growth response to cadaverine can be identified through exploration of the natural variation existing between plant accessions. In one study, ten *Arabidopsis* accessions were tested on cadaverine-containing media, showing accession-specific cadaverine responses for primary root growth, skewing, waving, and lateral root number (Strohm et al., 2015). Using a Quantitative Trait Loci (QTL) approach, the authors identified *ORGANIC CATION TRANSPORTER* 1 (*OCT1*) as contributing to the variation between the L*er* and Cvi accessions (Strohm et al., 2015). *OCT1* encodes a membrane-associated protein that was previously implicated in carnitine transport (Vaz and Wanders, 2002; Lelandais-Brière et al., 2007). Null *oct1* mutants were shown to exhibit a hypersensitive root-growth response to cadaverine (Strohm et al., 2015), suggesting a role for OCT1 in cadaverine eﬄux (Sagor et al., 2016; see above). This important result suggests that the natural variation existing between plant populations can be explored for identification of genes involved in the cadaverine-response pathway.

The inducing effects of cadaverine on root branching are equally interesting. Lateral root development is a complex, auxin-dependent process that has been shown to involve multiple phases, from initiation at the pericycle and primordium development to emergence from the primary root and subsequent growth (reviewed in Lavenus et al., 2013). Effort is needed to determine how cadaverine affects lateral root formation, the phases of lateral root development it acts upon, and to establish possible connections between cadaverine and hormone signaling.

In addition to modulating root-system architecture, cadaverine has also been shown to affect several aspects of shoot development, including promoting hypocotyl elongation in soybean (Campestre et al., 2011) and altering the number of epidermal cells and stomata morphology in leaves of barley (Çavuşoǧlu et al., 2007). Cadaverine has also been tied to plant reproduction; its content spiked in *Polianthes tuberosa* corms during floral initiation and then declined before floral development, suggesting a role in floral initiation (Huang et al., 2004).

## Cadaverine May Contribute to Environmental Stress Response

As with other polyamines, cadaverine has been implicated in stress response. However, there is a dichotomy between cadaverine acting as a stress protectant or exacerbating stress damage.

Cadaverine has been reported to facilitate seed germination and seedling growth under environmental stress. For instance, mustard seeds (*Brassica juncea* L.) exposed to salt, lead or cadmium displayed increased germination rate when treated with cadaverine, suggesting a role for this diamine in stress mitigation (Tomar et al., 2013b). Similarly, in barley (*Hordeum vulgare* L.), exogenous cadaverine promoted seed germination and seedling growth in the presence of salt (Çavuşoǧlu et al., 2007).

Cadaverine was reported to accumulate in the tissues of several plant species in response to a wide variety of environmental stimuli (Aziz et al., 1998; Kuznetsov et al., 2007; Cassán et al., 2009; Sziderics et al., 2010; Simon-Sarkadi et al., 2014). For instance, in the common ice plant (*Mesembryanthemum crystallinum L.*), cadaverine accumulated in response to heat shock, salt stress, and exogenous ethylene treatment (Shevyakova et al., 2001; Kuznetsov et al., 2007). Furthermore, local application of heat shock to either shoots or roots promoted cadaverine accretion in distal organs, suggesting transport throughout the plant (Shevyakova et al., 2001).

Similarly, pepper plants (*Capsicum annuum* L.) were shown to accumulate cadaverine and putrescine in leaves, and spermidine and spermine in roots, upon exposure to drought conditions (Sziderics et al., 2010). In leaves, polyamines may contribute some protective effect against water-deficient conditions by inhibiting potassium influx into guard cells, thereby inducing stomatal closure and reducing water loss (Liu et al., 2000).

While the previous studies reported cadaverine-induced stress mitigation, an experiment with *Arabidopsis thaliana* suggested induction of stress hypersensitivity. In this experiment, seedlings were pretreated with cadaverine for 1 week, and then moved to media containing 150 mM NaCl for another week. Cadaverine-pretreated seedlings displayed a hypersensitive response to salt despite an obvious accumulation of spermine, a polyamine previously associated with salt-stress mitigation (Yamaguchi et al., 2006; Liu et al., 2014). This result was interpreted to suggest that increased spermine levels, *per se*, may not be sufficient for salt-stress mitigation. Instead, an increase in spermine catabolic products may be required (Liu et al., 2014).

The previous discussion nicely illustrates a major complication in the study of cadaverine’s role in plant stress response: many environmental stimuli, such as cold, salt, and drought stresses, also influence the expression of putrescine-derived polyamine biosynthetic enzymes (reviewed in Alcázar et al., 2010). Furthermore, cadaverine is also known to influence the accumulation of putrescine-derived polyamines in plant tissues (Liu et al., 2014). Therefore, the contribution of cadaverine to plant stress response cannot be assessed in isolation. Instead, it will be important to carefully examine its impact in relation to that of putrescine-derived polyamines under the same conditions. Identification and characterization of additional polyamine response mutants will undoubtedly help in this difficult endeavor.

To further assess cadaverine’s role in plant-stress response, it will be critical to elucidate the pathways that lead to its biosynthesis, conjugation, transport and catabolism in control and stressful conditions. In species lacking clear *LDC* genes, such as *Arabidopsis* and rice, it will be necessary to investigate possible alternative pathway(s) for stress-induced cadaverine synthesis or uptake. In this regard, cadaverine delivery by rhizosphere and phyllosphere microbes should be considered. A more global analysis of the types of cadaverine-producing microbes associated with various plant species would be useful, as would an evaluation of the amount of cadaverine they deliver to the plant under diverse conditions. Furthermore, characterization of plant stress-response in the presence of cadaverine-defective microbial mutants should help demonstrate a role for microbial-derived cadaverine in stress mitigation.

## Conclusion and Future Prospects

Through endogenous synthesis or environmental uptake, cadaverine induces morphological changes that modulate plant development and environmental stress responses. This minireview is meant to provide a concise summary of the current knowledge of cadaverine’s role in plants, and is not an exhaustive review of relevant literature in the field.

While the molecular mechanisms of cadaverine action remain elusive, current literature has highlighted how it is produced, taken up, and metabolized in a few plant species. Research has also documented a role for cadaverine in plant growth, development and stress response. These data offer new avenues for increasing crop yield and engineering stress-tolerant plants.

Unfortunately, large gaps remain in our understanding of plant responses to cadaverine. To date, only a few genes contributing to cadaverine metabolism or response have been characterized. Additional gene identification and functional characterization will be critical to better understand cadaverine function in plants. In this context, recent technological developments in genetics, systems biology and genome editing offer amazing opportunities to unravel cadaverine-response pathways and related gene interaction networks.

Ultimately, expanding beyond current experimental setups that mostly investigate plant monocultures to also incorporate cadaverine-producing microbes, will be critical to evaluate the contribution of cadaverine in bidirectional plant-microbe interactions and their potential roles in environmental perception and stress responses.

Finally, human health will also benefit from a deeper understanding of these pathways, beyond its effects on crop improvement for food, feed and fiber production. Indeed, quinolizidine alkaloids are already used as pharmaceuticals to treat central nervous system disorders, addiction, and Alzheimer’s disease, and could serve as excellent starting points in the design of additional compounds for use in the treatment of multiple ailments.

## Author Contributions

AJ and NG contributed equally to this work. They wrote sections of the manuscript, then contributed to its editing and final formatting. AJ contributed **Figure 1**; NG contributed **Table 1**. PM contributed to editing materials written by AJ and NG, and final integration of the various sections.

## Conflict of Interest Statement

The authors declare that the research was conducted in the absence of any commercial or financial relationships that could be construed as a potential conflict of interest.
